# Dynamics of phase oscillator networks with synaptic weight and structural plasticity

**DOI:** 10.1038/s41598-022-19417-9

**Published:** 2022-09-02

**Authors:** Kanishk Chauhan, Ali Khaledi-Nasab, Alexander B. Neiman, Peter A. Tass

**Affiliations:** 1 Department of Physics and Astronomy, Ohio University, Athens, OH 45701 USA; 2 Neuroscience Program, Ohio University, Athens, OH 45701 USA; 3 Department of Neurosurgery, Stanford University, Stanford, CA 94305 USA

**Keywords:** Computational biophysics, Biophysics, Biophysical models, Dynamical systems, Network models, Neuroscience, Computational neuroscience, Neural circuits, Synaptic plasticity

## Abstract

We study the dynamics of Kuramoto oscillator networks with two distinct adaptation processes, one varying the coupling strengths and the other altering the network structure. Such systems model certain networks of oscillatory neurons where the neuronal dynamics, synaptic weights, and network structure interact with and shape each other. We model synaptic weight adaptation with spike-timing-dependent plasticity (STDP) that runs on a longer time scale than neuronal spiking. Structural changes that include addition and elimination of contacts occur at yet a longer time scale than the weight adaptations. First, we study the steady-state dynamics of Kuramoto networks that are bistable and can settle in synchronized or desynchronized states. To compare the impact of adding structural plasticity, we contrast the network with only STDP to one with a combination of STDP and structural plasticity. We show that the inclusion of structural plasticity optimizes the synchronized state of a network by allowing for synchronization with fewer links than a network with STDP alone. With non-identical units in the network, the addition of structural plasticity leads to the emergence of correlations between the oscillators’ natural frequencies and node degrees. In the desynchronized regime, the structural plasticity decreases the number of contacts, leading to a sparse network. In this way, adding structural plasticity strengthens both synchronized and desynchronized states of a network. Second, we use desynchronizing coordinated reset stimulation and synchronizing periodic stimulation to induce desynchronized and synchronized states, respectively. Our findings indicate that a network with a combination of STDP and structural plasticity may require stronger and longer stimulation to switch between the states than a network with STDP only.

## Introduction

Networks with adaptive coupling are used as models of various real-world systems, such as social^1^, chemical^2^, and neuronal networks^3–5^. Adaptive networks have also been used to study swarm dynamics^6^, epidemic spreading^7^, and optimization of power grids^8^. While system-specific models can be used for the system entities (nodes of the network) to study the dynamics of such networks, a phase oscillator model applies to several different network types and has been widely used due to its simplicity and tractability^9^. Networks of oscillators with frozen structure but adaptive coupling show the emergence of coherent, incoherent, and clustered states depending on the adaptation rules and may exhibit multistability^4,10–12^. With specific rules of adaptation, the degree of both global and clustered synchrony can be enhanced^13^. The rewiring of a network could also increase the level of synchrony through activity-dependent re-organization of the network^14^.

In neuronal networks, the adaptive (plastic) nature of the synaptic contacts is linked to normal brain function, learning of new skills and retention of long-term memories^15–17^, and formation of non-random and clustered assemblies of neurons^15,18,19^. Notwithstanding, network plasticity may also engender pathological neuronal synchronization observed in several brain disorders, such as epilepsy and Parkinson’s disease (PD)^20–22^. On the contrary, in Alzheimer’s disease (AD), disease progression is linked to pathological desynchronization and decoupling of neuronal populations^23–27^.

One of the prominent plasticity mechanisms is spike-timing-dependent plasticity (STDP), whereby the weights of synaptic contacts can change depending on the relative spike timings of pre- and post-synaptic neurons^28–34^. In networks of oscillatory neurons, STDP may lead to the formation of multiple metastable states, such as coexisting attractors of synchronized and desynchronized states, which may represent pathological and normal states^12,35^. A proper stimulation can be used to shift the network from an attractor of a synchronized state to that of a desynchronized state, or vice versa. In PD, the desirable therapeutical effect is long-lasting desynchronization that can be achieved using e.g. Coordinated Reset (CR) stimulation that aims to desynchronize^35,36^ and decouple^37^ neurons in subthalamocortical networks. In AD, a sensory stimulus-induced re-synchronization of neuronal networks that restores the coherence of gamma-band oscillations and spike-gamma coupling is a promising novel therapeutic approach^23,25,27^.

Another form of plasticity called structural plasticity (SP), operating on a longer time scale compared to STDP, involves the addition and elimination (pruning) of the synaptic contacts, which could be activity-dependent^15,38^. For instance, the homeostatic SP maintains a background (homeostatic) level of activity of the neurons^38,39^ and is essential for stabilizing the activity of the neuronal networks^19,39^ by both scaling the synaptic weights^40^ and adding and removing contacts^15,41^. On the other hand, SP could lead to the stabilization of pathological conditions such as chronic pain, neuropathic pain, and nociceptive hypersensitivity^42^.

In modeling studies, several different implementations of STDP and SP with homeostatic mechanisms have been used to study specific brain functions^15^. Broadly, these studies either implement the addition and elimination of synapses besides the synaptic weight changes, or model synapse formation and elimination via dendritic and axonal remodeling and outgrowth^15^.

A major question is how the interplay of STDP and SP affects a network of oscillatory neurons and controls the collective dynamics of the network. This question is particularly relevant to the development of therapeutic stimulation techniques aimed at shifting the operational point of the collective neuronal dynamics from the pathological to physiological state. A recent computational study of a detailed model of basal ganglia^43^ showed that following the desynchronization CR stimulation, homeostatic SP may decrease the network connectivity, thereby slightly suppressing the neuronal synchrony in a stimulation-free setting. Consequently, the desynchronization effect of stimulation increases during the stimulation-free epoch^43^, as observed in clinical trials^44,45^.

The Kuramoto model of phase oscillators is extensively used for understanding a plethora of collective dynamics phenomena^46–49^. Here we implement a network of phase oscillator model neurons with SP and a standard additive STDP to study the collective dynamics that results from the interplay of these distinct adaptive mechanisms. In our model, the SP incorporates a stochastic pruning of existing synaptic contacts and the addition of new ones^50,51^ with a time scale separation of neuronal spiking, STDP, and SP as suggested by previous studies^15,52^. In the case of adaptive coupling only (STDP), the connectivity remains frozen while the contact strength can change. When SP is combined with STDP, both coupling and connectivity can change. We also examine the effects of a desynchronizing CR stimulus and a synchronizing periodic stimulus on the network in the two plasticity schemes.

## Methods

### Kuramoto model with STDP and SP

We consider *N* phase oscillators coupled on a random network. The $N\times N$ adjacency matrix ${\varvec{A}}$ sets the network’s structural connectivity, whereas the functional connectivity, i.e., the coupling strength between the connected oscillators, is set by the $N\times N$ weight matrix ${\varvec{W}}$. Elements of both these matrices are time-dependent, representing two slow adaptation processes: STDP for ${\varvec{W}}$ and SP for ${\varvec{A}}$. The phase of an oscillator is governed by

1 $$\begin{aligned} \displaystyle {\dot{\phi }}_i=\omega _i - N^{-1} \sum _{j=1}^N A_{i,j}(t) w_{i,j}(t) \sin (\phi _i-\phi _j) + S(\phi _i,t) + \xi _i(t), \quad i=1,..,N. \end{aligned}$$

where $\omega _i$ is the characteristic frequency of the $i$-th oscillator. For a network of identical oscillators, $\omega _i=\omega$ $\forall i$, while for a network of non-identical oscillators, $\omega _i$ can be sampled from a random distribution, such as Gaussian. The second term introduces the Kuramoto-type coupling^53^. The element, $A_{i,j}(t)$, of the adjacency matrix is either 1 if the contact from the oscillator *j* to *i* exists or 0 otherwise. The weight matrix element, $w_{i,j}(t)$, lies between 0 and the maximum allowable weight, $\gamma$, and gives the coupling strength of the contact from the oscillator *j* to *i*. $S(\phi _i,t)$ is the external stimulus whose action depends on the phase of the oscillator, as explained later, and $\xi _i(t)$ is the independent Gaussian white noise with the intensity *D* such that $\langle \xi _i(t)\xi _j(t')\rangle =2D \delta _{i,j}\delta (t-t')$.

We interpret each oscillator as a neuron eliciting a spike whenever its phase crosses an integer multiple of $2\pi$. Thus, in Eq. (1), the index *i* refers to a postsynaptic neuron while the index *j* marks a presynaptic neuron. The adjacency matrix element, $A_{i,j}(t)=1$, represents an existing synaptic contact from neuron *j* to *i* and the weight matrix element, $w_{i,j}(t)$, gives its synaptic weight.

In the following, we consider two kinds of plasticity that modify the coupling (synaptic weight) and the structure of the network, STDP and SP:

(i) STDP governs the functional connectivity, i.e., the evolution of the weight matrix according to the microscopic dynamics of the oscillators, ${\varvec{W}}={\varvec{W}}(t)$. STDP does not change the network structure, ${\varvec{A}}=\text {constant}$;
(ii) SP changes the network structure according to the dynamics of the synaptic weights and homeostatic mechanisms, ${\varvec{A}}={\varvec{A}}(t)$.

For a combination of STDP and SP (STDP + SP), both weight and adjacency matrices become time-dependent, ${\varvec{W}}={\varvec{W}}(t)$ and ${\varvec{A}}={\varvec{A}}(t)$.

### STDP

**Figure 1:**
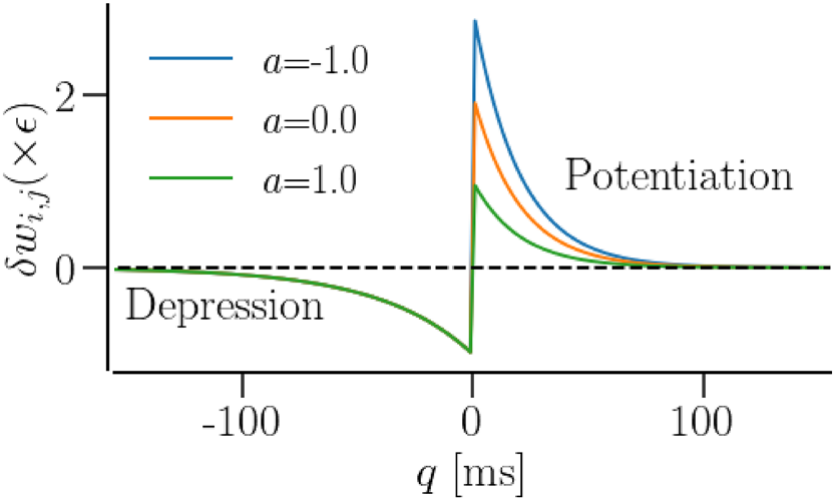
Spike-timing-dependent plasticity. Synaptic weight increment vs. spike timing difference (time lag) according to Eq. (2) for different values of the asymmetry parameter, *a*. When a postsynaptic neuron fires before or at the same time as its presynaptic partner, i.e., when $q \le 0$, the synaptic weight decreases (depression), while it increases when $q>0$ (potentiation). A positive asymmetry makes the STDP rule potentiation dominant, zero makes it symmetric, and negative makes it depression dominant.

The weight matrix evolves according to a standard additive rule: $w_{i,j}(t) \rightarrow w_{i,j}(t) + \delta w_{i,j}$, where the increment of each weight matrix element, $\delta w_{i,j}$, is determined by the time lag, *q*, between the latest spike times, $t_i$ and $t_j$, of the pre- and post-synaptic neurons, *i* and *j*, respectively^36,54^ (Fig. 1).

2 $$\begin{aligned} \delta w_{i,j}(q)= \varepsilon {\left\{ \begin{array}{ll} (b-a) \exp {\left( -\frac{|q|}{\tau _p}\right) }, &{} q > 0,\\ -\exp {\left( -\frac{|q|}{b\tau _p}\right) }, &{} q \le 0. \end{array}\right. } \end{aligned}$$

Here, $q=t_i-t_j$ and the parameter, $\varepsilon \ll 1$, ensures a longer time scale of synaptic weight change compared to the fast spiking dynamics of the neurons as suggested in Refs.^15,38^. $\tau _p$ is the time constant for long-term potentiation (LTP) and *b* scales the time constant of long-term depression (LTD) with respect to potentiation, $\tau _d=b\tau _p$. The total weight increment is $\Delta w_{i,j} = \int _{-\infty }^\infty \delta w(q) dq= -\varepsilon a \tau _p$. Thus, the parameter *a* in Eq. (2) determines the overall asymmetry of STDP: $a>0$ results in depression domination, $a <0$ in potentiation domination, and $a=0$ corresponds to the balanced STDP. At any time, the synaptic weights, $w_{i,j}$, remain within the bounds $[0,\gamma ]$. The above learning rule is implemented as a set of differential equations for the weight matrix, ${\varvec{W}}$, and traces $\{x(t), y(t)\}$ for pre- and post-synaptic spike trains^55^,

3 $$\begin{aligned} {\dot{w}}_{i,j}= & {} \varepsilon \left[ (b-a) x_j \,\delta (t-t_i) - y_i\, \delta (t-t_j)\right] , \nonumber \\ {\dot{x}}_j= & {} -\frac{1}{\tau _p} x_j + \sum _{\{t_j\}} \delta (t-t_j), \quad {\dot{y}}_i = -\frac{1}{b\tau _p} y_i + \sum _{\{t_i\}} \delta (t-t_i). \end{aligned}$$

The summations are carried over all spike times of pre- and post- synaptic neurons, $\{t_j\}$ and $\{t_i\}$, respectively.

### SP

We consider several experimental findings to model SP in the framework of a phase oscillator network. Cortical neurons may possess large dendritic arbors. If two neurons lie in the vicinity of one another, the axon of one may come close to the dendrite of the other at multiple locations to form potential synapses, some of which may develop into actual synaptic contacts^56,57^. Consequently, a pair of neurons either remains disconnected or develops several synaptic contacts^56,58,59^. Modeling studies suggest that multiple synaptic contacts between given neurons stabilize the neuronal network dynamics and underlie long-term memory storage^60,61^. Nonetheless, to simplify the calculations while investigating the (de)synchronization dynamics, we replace multiple synaptic contacts between two particular neurons by one contact that accounts for the overall effect of the activity of the presynaptic neuron on the postsynaptic partner.

The structural changes consist of the pruning of existing synaptic contacts and the addition of new ones. In our model, both of these processes are random and run on a time scale much longer than the average period of oscillations. Pruning or adding a synaptic contact changes the adjacency matrix, ${\varvec{A}}$. Pruning the existing contact from neuron *j* to *i* changes the corresponding element of the adjacency matrix, $A_{i,j}$, from 1 to 0, whereas adding a contact changes it from 0 to 1. Thus, each non-diagonal element of the adjacency matrix is modeled as a two-state stochastic process.

In the nervous system, neuronal activity and synaptic connectivity are closely linked, and different homeostatic mechanisms are implemented to maintain physiologically meaningful operating ranges for both structure and function^39,41,42,62–64^. For instance, SP with homeostatic mechanisms can maintain a target level of neuronal activity^19,38,39^. Homeostatic mechanisms can cause changes in intrinsic neuronal excitability^65^ and scaling of synaptic weights^39^ besides addition and pruning of contacts depending on the activity level of the neurons^66^.

**Figure 2:**
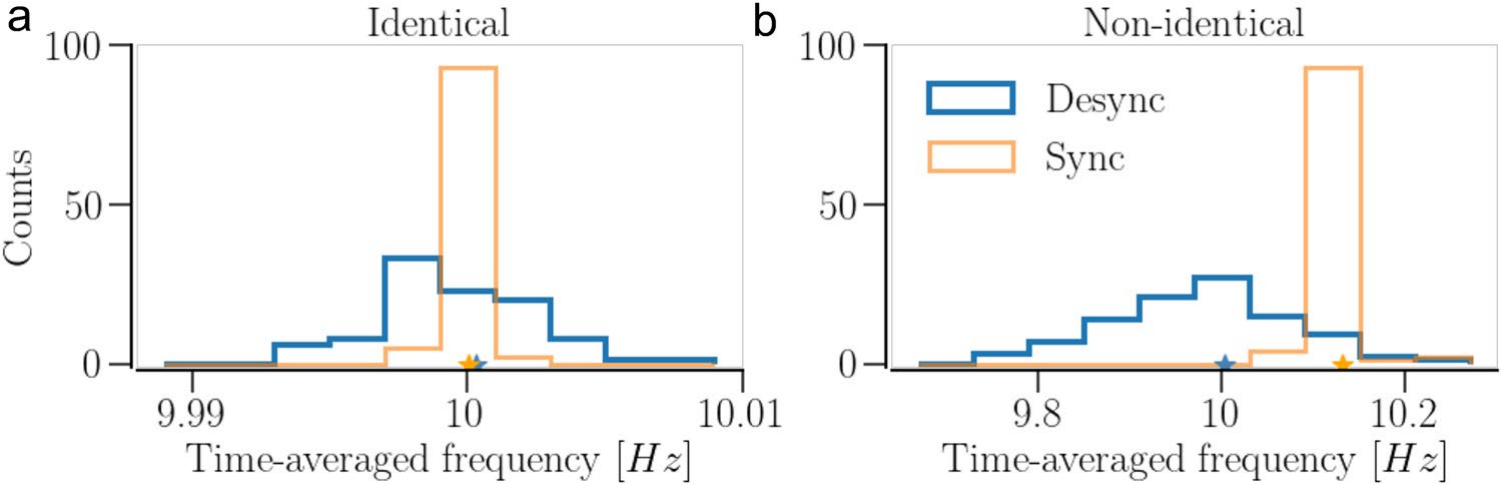
Time-averaged frequency distributions in the synchronized and desynchronized states of a network of $N=100$ oscillators with STDP. (**a**) and (**b**) show networks of identical and non-identical oscillators, respectively. The distribution averages (network-averaged frequencies) are marked by stars with corresponding colors. Parameters are $a = 0.3$ and $\gamma = 3$ for the identical oscillators; $a = 0.3$ and $\gamma = 15$ for the non-identical oscillators; the average node degree density (NDD), $\langle \beta \rangle$ is 0.2 for the identical, and 0.4 for the non-identical oscillator networks.

For the Kuramoto model with a single first Fourier mode coupling function used in this study, i.e., without the constant phase shift in the coupling term or additional cosine coupling term, cf.^67,68^, it is expected that the time-averaged frequencies of the oscillators in synchronized and desynchronized states do not vary significantly, as opposed to those in the states obtained with a coupling function with an additional cosine coupling term (or phase shift) and/or higher Fourier modes^67,68^. This is illustrated in Fig. 2 showing the distributions of the time-averaged frequencies of the oscillators in synchronized and desynchronized states of a network of identical oscillators with STDP only. Even in the case of non-identical oscillators, the network-averaged frequency shift is less than 2%, see Fig. 2b. Consequently, a homeostatic SP model that responds to the neurons’ firing rates in order to keep them in a target firing range is not employed in this study. Instead, our SP model mechanism takes into account that synaptic connectivity is fundamentally constrained for a number of reasons, including limited axonal space available for synapses, limited anatomical overlap of neuronal arbors as well as anatomical and metabolic constraints of synapse formation^62–64,69^. Such constraints on synaptic connections vary depending on the neuron type and brain region^63^. A detailed model that includes a spatial network structure, among many other details, would be required to account for the experimental findings on, e.g., barrel cortex^70^. Our phase oscillator network model lacks spatial dimension. Thus, abstracting from details, our homeostatic SP rule imposes bounds on each neuron’s incoming node degree density (In-NDD), defined by

4 $$\begin{aligned} \displaystyle \beta _i(t)=\frac{1}{N} \sum _{j=1}^N A_{i,j}(t), \end{aligned}$$

such that $\beta _i \in [\beta _\text {min}, \beta _\text {max}]$. This precludes the physiologically non-typical or impossible situations of completely disconnected neurons or hyperactive ones with a large number of presynaptic partners. In our model of SP, the addition is a homeostatic process, and the pruning has both homeostatic and weight-dependent components. The addition rate, $\Lambda _\text {add}$, decreases with increase in $\beta _i$ and vanishes when $\beta _i$ exceeds its maximal value, $\beta _\text {max}$. Similarly, the homeostatic pruning rate, $\Lambda _\text {prn}^H$, increases with $\beta _i$ saturating near $\beta _\text {max}$, irrespective of the synaptic weight of the contact. If a contact’s weight becomes small enough and the neuron has more than the minimum allowed number of contacts [$(N-1) \beta _\text {min}$], it can also be pruned at a faster weight-dependent rate, $\Lambda _\text {prn}(\beta _i,w_{i,j})$^61,71^. We do not put any constraints on the number of postsynaptic partners or the outgoing node degree density (Out-NDD) of the neurons, defined by

5 $$\begin{aligned} \displaystyle \beta _j^\text {out}(t) = \frac{1}{N} \sum _{i=1}^N A_{i,j}(t). \end{aligned}$$

Pruning of an existing synaptic contact (with $A_{i,j}=1$) occurs at random, either at a rate determined by the synaptic weight, $w_{i,j}$, and the In-NDD, $\beta _i$, or at a lower homeostatic rate regardless of the synaptic weight.

6 $$\begin{aligned} \displaystyle \Lambda _\text {prn}(\beta _i,w_{i,j})= & {} \lambda _0\ g(\beta _i,{\widetilde{\beta }}_\text {min},\nu )[1-g(w_{i,j},W_\text {min},\nu )] ,\nonumber \\ \Lambda _\text {prn}^H(\beta _i)= & {} \eta \lambda _0 g(\beta _i,{\widetilde{\beta }}_\text {max},\nu ), \end{aligned}$$

where $\lambda _0$ is the maximum rate of weight-dependent pruning, and $g(x,x_0,\nu )$ is the logistic function,

$$\begin{aligned} \displaystyle g(x,x_0,\nu ) = \left[ 1+e^{-\frac{(x-x_0)}{\nu x_0}} \right] ^{-1}. \end{aligned}$$

The first equation in (6) establishes the lower bound of In-NDD by ensuring the pruning of predominantly weak contacts: $W_\text {min}$ is the weight below which a synaptic contact is most likely to be pruned if the In-NDD is above $\beta _\text {min}$. The second equation in (6) implements the homeostatic pruning on a longer time scale with the rate scaled by the factor $\eta$ with respect to the weight-dependent pruning, if a neuron’s In-NDD approaches or exceeds $\beta _\text {max}$. ${\widetilde{\beta }}_\text {min,max}$ are related to $\beta _\text {min,max}$ as discussed below. The parameter $\nu$ in the logistic function determines its steepness.

The addition of a new synaptic contact from neuron *j* to *i* (with $A_{i,j}=0$) occurs randomly at the rate $\Lambda _\text {add}$ that depends on the *i*-th neuron’s In-NDD, $\beta _i$,

7 $$\begin{aligned} \displaystyle \Lambda _\text {add}(\beta _i) = \eta \lambda _0\, [1-g(\beta _i,{\widetilde{\beta }}_\text {max},\nu )]. \end{aligned}$$

Conventionally, $\eta \ll 1$, as the addition of new contacts occurs on a longer time scale compared to the weight-dependent pruning^60^. The weight for a newly established contact is sampled from a uniform distribution in the range $[0, 0.05\gamma ]$ as the new contacts are likely to be weaker than the existing ones^72^.

**Figure 3:**
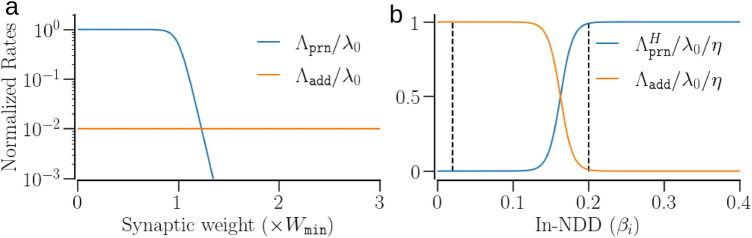
Structural plasticity rates as functions of the synaptic weight and the In-NDD of a neuron. (**a**) The maximum rates of pruning and addition (Eqs. 6 and 7) normalized with respect to $\lambda _0$ vs. the synaptic weight. The addition rate is shown for comparison for $\eta =0.01$. (**b**) The rates of homeostatic addition and pruning normalized with respect to $\lambda _0 \times \eta$ vs. the In-NDD of a neuron. The vertical lines lie at the $\beta _\text {min}=0.02$ and $\beta _\text {max}=0.2$. The addition rate drops to $\approx 0$ for $\beta _i > \beta _\text {max}$ and the pruning rate increases to the maximum for $\beta _i > \beta _\text {min}$. Parameters for (**a**): $\beta =1$ for pruning, and $\beta =0$ for addition.

To correct for the possible undershooting of node degree due to pruning and overshooting due to addition, the midpoints of the logistic function were corrected as follows. For addition, ${\widetilde{\beta }}_\text {max}=\beta _\text {max}/(1-\nu \log p_+)$, with $p_+=1/(\eta N^2)$. For pruning, ${\widetilde{\beta }}_\text {min}=\beta _\text {min}/(1+\nu \log p_-)$, with $p_-=1/N^2$. Figure 3 illustrates the maximum rates of pruning and addition.

We observed no relevant variation in the time-averaged frequency distribution and the network-averaged frequency in the synchronized and desynchronized states of a network with STDP + SP compared to those for a network with STDP only (cf. Fig. 2).

### Parameters and numerical simulation

We choose the parameters such that the network can settle either in a synchronized or a desynchronized state in the absence of SP. For a network of non-identical oscillators, we draw the natural frequencies of the oscillators, $\omega _i$, from a Gaussian distribution, $\omega _i=2\pi f_0 (1+\sigma _f \zeta _i)$, where $f_0$ is the mean frequency, $\sigma _f$ is the normalized standard deviation (SD) and, $\zeta _i$ is a zero-mean Gaussian number with a unit SD. The mean frequency, $f_0$, is set to 10 Hz, giving the average period of oscillations, $T_0=0.1$ s. The normalized SD of the natural frequency, $\sigma _f$, is set to 0.01. For an identical oscillator network, $\omega _i=2\pi f_0 \forall i$. The intensity of the white noise source in Eq. (1) is taken to be $D=0.1$.

The parameters for the STDP (Eq. 2) and SP (Eqs. 6 and 7) are as follows: the STDP potentiation time constant, $\tau _p = 0.02$ s, the scaling parameter, $b=2$, and the depression time constant, $\tau _d=b\tau _p=0.04$ s. The asymmetry parameter, *a*, is varied while $\varepsilon$ is set to $10^{-3}$. SP operates on a time scale longer than that of STDP. In our model, the time scale of SP is set by the maximal weight-dependent pruning rate, $\lambda _0$, which is varied. Unless otherwise specified, we set $\lambda _0=1/(60000 T_0)=1.667\times 10^{-4}$ s$^{-1}$ and the scaling parameter $\eta =0.01$ in the simulations, so that the maximal addition rate is $1.667\times 10^{-6}$ s$^{-1}$. The cutoff value of weights for pruning in Eq. (6) is set to $W_\text {min}= \gamma \times 10^{-2}$. The In-NDD bounds are $\beta _\text {min}=0.02$ and $\beta _\text {max}=0.2$, and the slope parameter, $\nu$, of the logistic function is set to 0.05.

The longest time scale in the model is associated with homeostatic addition and pruning with the characteristic time of $1/(\eta \lambda _0) \sim 10^6$ s. The time scale of weight-dependent pruning is $1/\eta$ times shorter than that of homeostatic addition and pruning, but still much longer than the average oscillation period. Because of such diverse time scales, numerical simulations are carried out in two sequential steps. First, the set of equations (1, 3) describing the microscopic dynamics of the oscillators along with STDP are integrated during the time $T_0 \ll {\mathscr {T}} <1/\lambda _0$ with the time step of 0.002 s. During that time the adjacency matrix remains frozen. Second, the structural update described in the above section is implemented resulting in an updated adjacency matrix. We use ${\mathscr {T}}=300$ s. Thus, the structural updates are performed every 3000 average periods, ${\mathscr {T}}=3000T_0$. Shorter windows with ${\mathscr {T}}<300$ s do not change the results but lengthen the calculations.

Initial conditions are as follows. Phases of oscillators are uniformly distributed between 0 and $2\pi$. The initial connectivity is set by a random Erdos-Renyi graph with the probability $p_\text {con}$. The initial values for weights, $w_{i,j}$, are drawn from a uniform distribution, $[W_0-\Delta _W,W_0+\Delta _W]$, where $W_0$ is the initial mean weight and $2\Delta _W=0.1$ is the spread of weights about the mean.

In this paper, we present the results for networks of $N=100$ oscillators. The larger networks of $N=200$ and 500 oscillators demonstrate qualitatively similar dynamics. All network simulations include STDP, meaning that the weights are always plastic. The networks with SP have STDP + SP and the networks without SP have STDP only.

### Measures of collective dynamics

The degree of synchronization in the network is characterized by the Kuramoto order parameter^9^,

8 $$\begin{aligned} \displaystyle {\mathscr {R}}(t) = \frac{1}{{\mathscr {T}}} \int _{t-{\mathscr {T}}}^t dt'\left| \frac{1}{N} \sum _{m=1}^N e^{\iota \phi _m(t')}\right| , \end{aligned}$$

where $\phi _m(t)$ is the phase of $m$-th neuron. ${\mathscr {R}}(t)$ varies between 0 (absence of in-phase synchronization) and 1 (perfect in-phase synchronization).

The synaptic weight dynamics of the network is given by the distribution of the synaptic weights and the normalized mean synaptic weight of the network, $\langle W\rangle (t)$, defined as

9 $$\begin{aligned} \displaystyle \langle W\rangle (t) = \frac{1}{N} \sum _{i=1}^{N} W_i(t), \quad W_i(t) = \frac{1}{k_i \gamma } \sum _{j=1}^{N} A_{i,j}(t) w_{i,j}(t), \end{aligned}$$

where $k_i$ is the node degree (i.e., number of presynaptic partners) of the $i-$th neuron. $\langle W\rangle \rightarrow 1$ (0) is typically related to synchrony (desynchrony).

The dynamics of the network structure is quantified by the In-NDD distribution and the mean In-NDD of the network,

10 $$\begin{aligned} \displaystyle \beta (t) = (1/N) \sum _{i=1}^N \beta _i(t). \end{aligned}$$

### Stimulation

**Figure 4:**
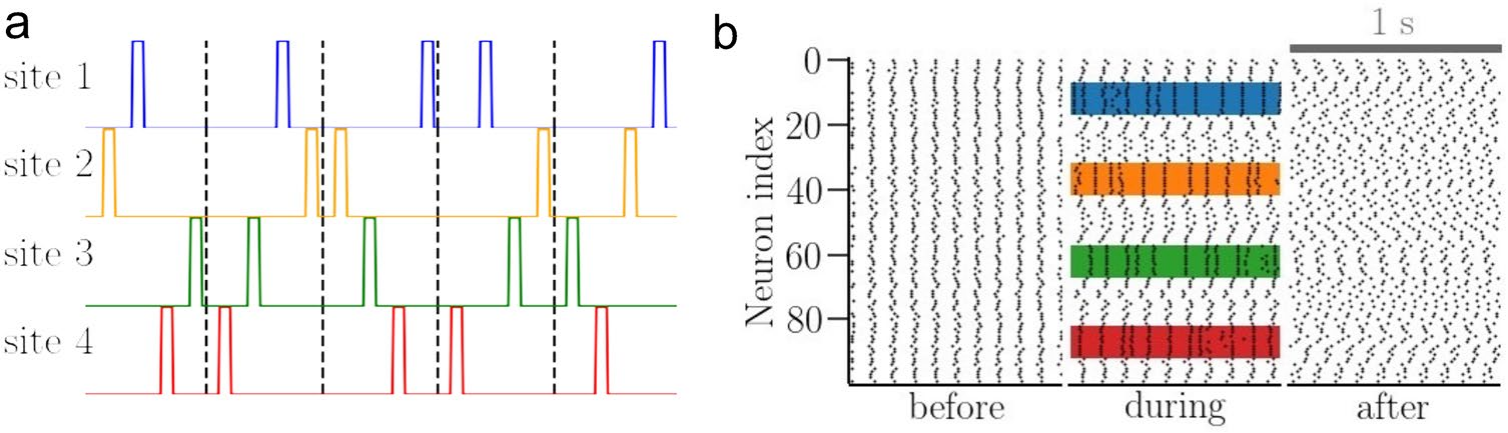
Rapidly varying sequences (RVS) CR stimulus and its effect on a synchronized network of identical oscillators. (**a**) Five cycles of RVS CR stimulus, separated by vertical dashed lines, delivered at $N_c=4$ sites (color coded). (**b**) Raster plots before, during, and 10 h after stimulation. Stimulation sites are indicated by colored patches. Parameters are $a=0.3$, $\gamma =3$; stimulus: $I_s=400$, duration = 70 min.

The dynamics and structure of neuronal networks can be manipulated by stimulation^35,37,43,54,73–83^. We use an anti-kindling coordinated reset (CR) stimulation to desynchronize initially synchronized networks. CR stimulus is administered to $N_c$ sites in a population of *N* oscillators, as described in^35,54,84^. Briefly, in CR stimulation with fixed sequences, one cycle of period $T_s$ consists of $N_c$ pulses of amplitude $I_s$ and width $t_\text {pulse}$. These $N_c$ pulses are administered to $N_c$ sites (i.e., one pulse per site) in a fixed order at intervals $T_s/N_c$ during each cycle, thus, stimulating one site with one pulse per cycle. The order in which the $N_c$ pulses are delivered to the $N_c$ sites is called the sequence. In CR stimulation with rapidly varying sequences (RVS CR)^54,85^, used in this study, the order in which the sites are stimulated is changed randomly for every stimulation cycle as illustrated in Fig. 4a. To synchronize an asynchronous network, we used a kindling stimulus^35,54^ in which $N_c$ sites are stimulated simultaneously.

The stimulus, $S(\phi _i,t)$, in Eq. (1) is phase-dependent^84^, $S(\phi _i,t) = X_i(t) I_s \cos \phi _i$, where $I_s$ is the stimulus intensity and $X_i(t)$ is the sequence of unit-magnitude pulses. In the following, we considered $N_c=4$ sites with *N*/10 oscillators within each site. The single pulse width was set to $t_\text {pulse}=10$ ms, and the stimulus cycle period, $T_s=T_0=0.1$ s. Figure 4b illustrates the effect of RVS CR stimulation on a network of synchronized oscillators. The raster plot before stimulation shows the network synchrony. During stimulation, the oscillators remain synchronized within stimulated sites (four colored patches) while the rest of the oscillators run out of synchrony. The network attains a desynchronized state after stimulation.

## Results

Previous studies showed that recurrent neuronal networks with STDP unbalanced towards depression possess bistability with co-existent attractors corresponding to weak and strong synaptic weights^86^. When individual neurons fire periodically, such bistability involves co-existent synchronized and desynchronized states. A proper stimulation could shift the network from a synchronized state to a desynchronized state, resulting in a long-term desynchronization^35–37,75,78,87,88^, or from a desynchronized to a synchronized state^23,25,27^. We hypothesize that the presence of SP may enhance the effect of STDP. On the one hand, pruning removes predominantly weaker synaptic contacts, thereby decreasing the overall connectivity. On the other hand, the addition of new contacts between previously disconnected neurons, which may get potentiated further, would favor synchronization.

### Asymptotic dynamics

We determine the spontaneous asymptotic states by setting the external stimulus, $S(\phi _i,t)=0$ in Eq. (1) for the networks of identical and non-identical oscillators with two schemes of plasticity: STDP only and STDP + SP. The states are characterized by the order parameter, ${\mathscr {R}}(t)$, mean synaptic weight, $\langle W\rangle (t)$, and mean In-NDD, $\beta (t)$. The states form attractors since the measures mentioned above converge to values corresponding to one of the asymptotic states. Thus, we determine the basins of attraction of the asymptotic states with respect to the initial mean In-NDD, $\beta (0)$, and mean synaptic weight, $\langle W\rangle (0)$.

**Figure 5:**
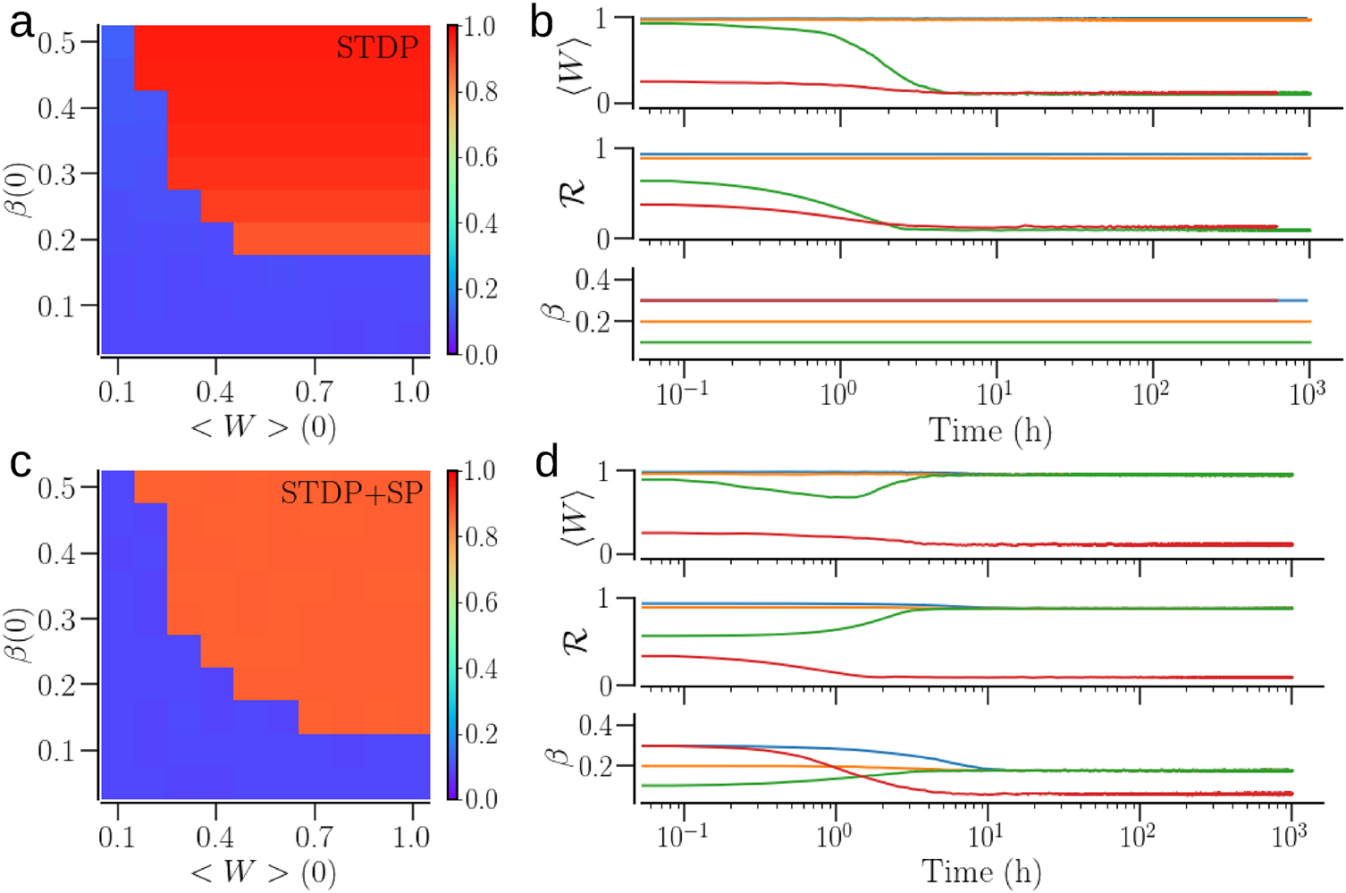
The asymptotic states for a network of identical oscillators (with $\omega _i=2\pi f_0$) and their basins of attraction in the two plasticity schemes considered—STDP and STDP + SP. (**a**,**b**) Refer to a network with STDP only; panels (**c**,**d**) correspond to the network with STDP + SP. The colors in (**a**) and (**c**) indicate the values of the order parameter, ${\mathscr {R}}$, in the asymptotic states when the system begins with the initial mean synaptic weight and In-NDD given by $\langle W\rangle (0)$ and $\beta (0)$. (**b**) and (**d**) show examples of time evolution of the mean synaptic weight, $\langle W\rangle$, the order parameter, ${\mathscr {R}}$, and the mean In-NDD, $\beta$, for different initial conditions (color coded) as the network transitions to a synchronized or desynchronized state, for STDP and STDP + SP, respectively. Parameters are $a=0.3$, $\gamma =3$.

We first consider a network of identical oscillators. Figure 5 shows that for both plasticity schemes, the network settles in either a synchronized or a desynchronized state depending on the initial conditions. An initial state with weak synaptic contacts or with sparse structure results in a desynchronized state (blue area in Fig. 5a,c). The desynchronized state is characterized by small values of the mean synaptic weight and order parameter for both the plasticity schemes (Fig. 5b,d). With SP, the contacts are prone to pruning, the weak ones more than the others. Also, the newly added contacts are given small initial weight, and thus, are likely to get depressed and removed in a desynchronized state. As a result, the network becomes extremely sparse with the final mean In-NDD close to the minimum, $\beta _\text {min}$, in a desynchronized state. The red curve in Fig. 5d demonstrates this for an initial condition with relatively large initial mean In-NDD, $\beta (0)=0.3$, but small initial mean synaptic weight, $\langle W\rangle (0)$, leading to a desynchronized state with a small mean synaptic weight, order parameter, and mean In-NDD.

Synchronization, on the other hand, promotes potentiation of the synaptic contacts, resulting in large values of order parameter and mean synaptic weight. In the presence of SP, some new contacts between the previously disconnected oscillators can appear and get potentiated, allowing for the accumulation of contacts over time. The number of incoming contacts is bounded by the maximum allowed In-NDD, $\beta _\text {max}$. As a result, the average In-NDD approaches $\beta _\text {max}$ in the synchronized state. Furthermore, a visual comparison of basins of attraction in Fig. 5a,c confirms an increase in the size of the basin of attraction of the synchronized state due to SP, showing that the synchronized state can be achieved with smaller initial connectivity for the given initial mean synaptic weight in the presence of SP. With $\beta (0) > \beta _\text {max}$, the network with STDP only can get more strongly synchronized than the one with STDP + SP since the In-NDD remains fixed in the absence of SP, ie., $\beta (t)=\beta (0)$, while it reduces to $\le \beta _\text {max}$ with SP.

**Figure 6:**
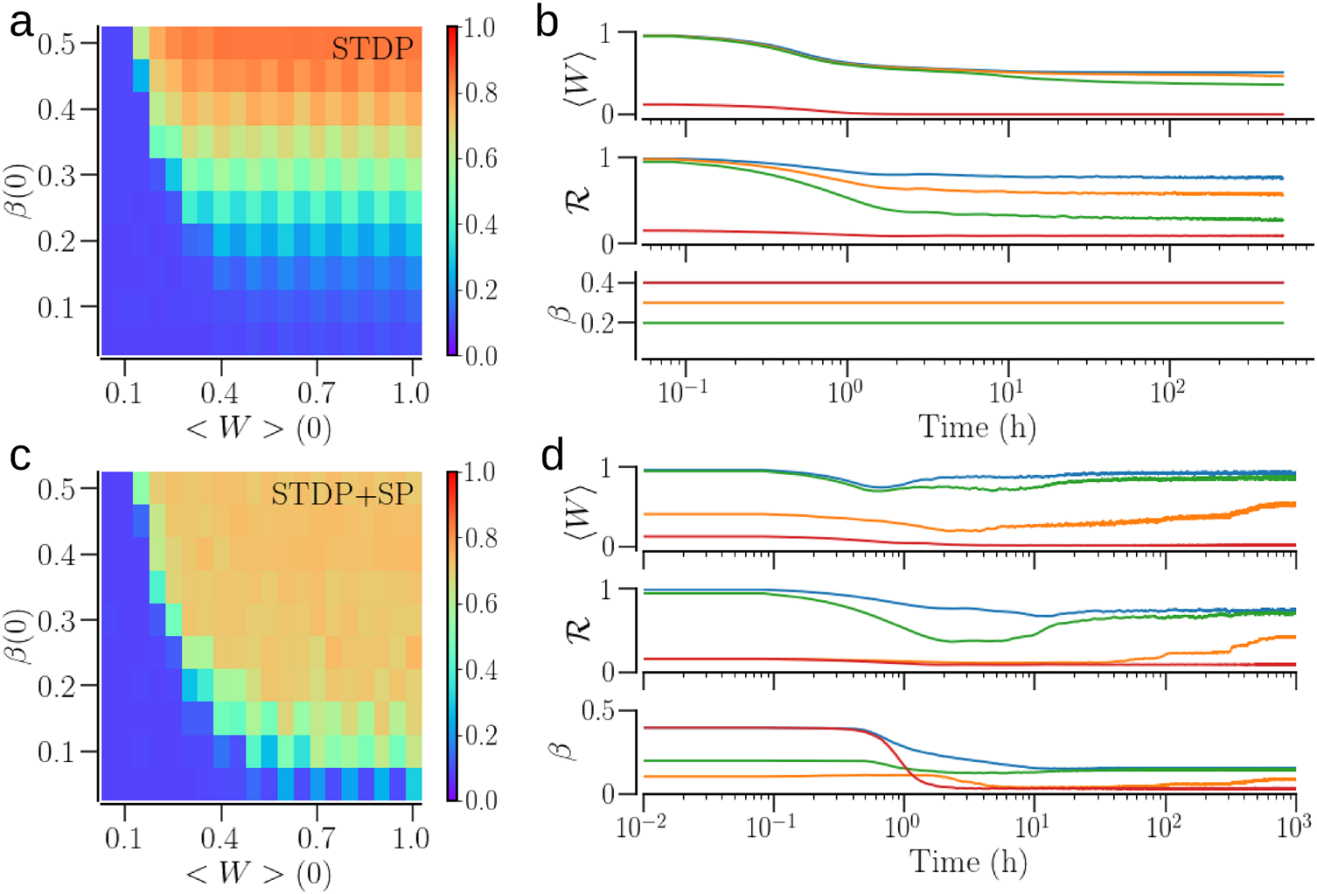
The asymptotic states for a network of non-identical oscillators and their basins of attraction in the two plasticity schemes. The natural frequency of oscillators, $\omega _i$, were sampled from the Gaussian distribution. (**a**,**b**) Refer to a network with STDP only; (**c**,**d**) corresponds to the network with STDP + SP. The colors in (**a**) and (**c**) indicate the values of the order parameter, ${\mathscr {R}}$, in the asymptotic states when the system begins with the initial mean synaptic weight and In-NDD given by $\langle W\rangle (0)$ and $\beta (0)$. (**b**) and (**d**) Show examples of time evolution of the mean synaptic weight, $\langle W\rangle$, the order parameter, ${\mathscr {R}}$, and the mean In-NDD, $\beta$ for different initial conditions (color coded) as the network transitions to a synchronized, desynchronized, or intermediate state, for STDP and STDP + SP, respectively. Parameters are $a=0.3$, $\gamma =15$.

A network of non-identical oscillators may settle in partially synchronized states with an order parameter much smaller than 1. This is illustrated by a spread of colors in Fig. 6a,c showing the maps of the order parameter vs. the initial conditions. We classify the states with the order parameter $0.2<{\mathscr {R}}<0.7$ as partially synchronized, exemplified by the time traces of the mean synaptic weight and order parameter in Fig. 6b,d. The frequency difference of the connected oscillators significantly affects the synaptic weights of the contacts: the weights of those from the faster oscillators to the slower ones increase while the opposite decrease in strength^35^. In the presence of SP, the depressed contacts get pruned, reducing the node degree. At the same time, the addition of new contacts from the faster to the slower oscillators can enhance synchronization as those are likely to get potentiated. This way, the interplay of STDP and SP leads to synchronized states with lesser structural connectivity (i.e., increased sparseness of contacts), compared to a network with STDP only. This is reflected by the increase in the size of the basin of attraction of the synchronized state for the network with STDP + SP in Fig. 6c compared with the network with STDP only, shown in Fig. 6a.

The existence of bistability depends on the asymmetry parameter, *a*, of the STDP rule (Eq. 2). An increase in *a* makes the STDP rule more depression dominant, and thus, promotes weakening of the synaptic contacts and ultimately desynchrony. On the contrary, a decrease in *a* makes the STDP less depression dominant or potentiation dominant, supporting synchrony. Consequently, if *a* is sufficiently large (small), the network with STDP only settles in globally desynchronized (synchronized) states, and the bistability exists for intermediate values of $a \in (0.15~~0.6)$. We observed that the inclusion of SP insignificantly affects the range of the asymmetry parameter where the bistability exists.

**Figure 7:**
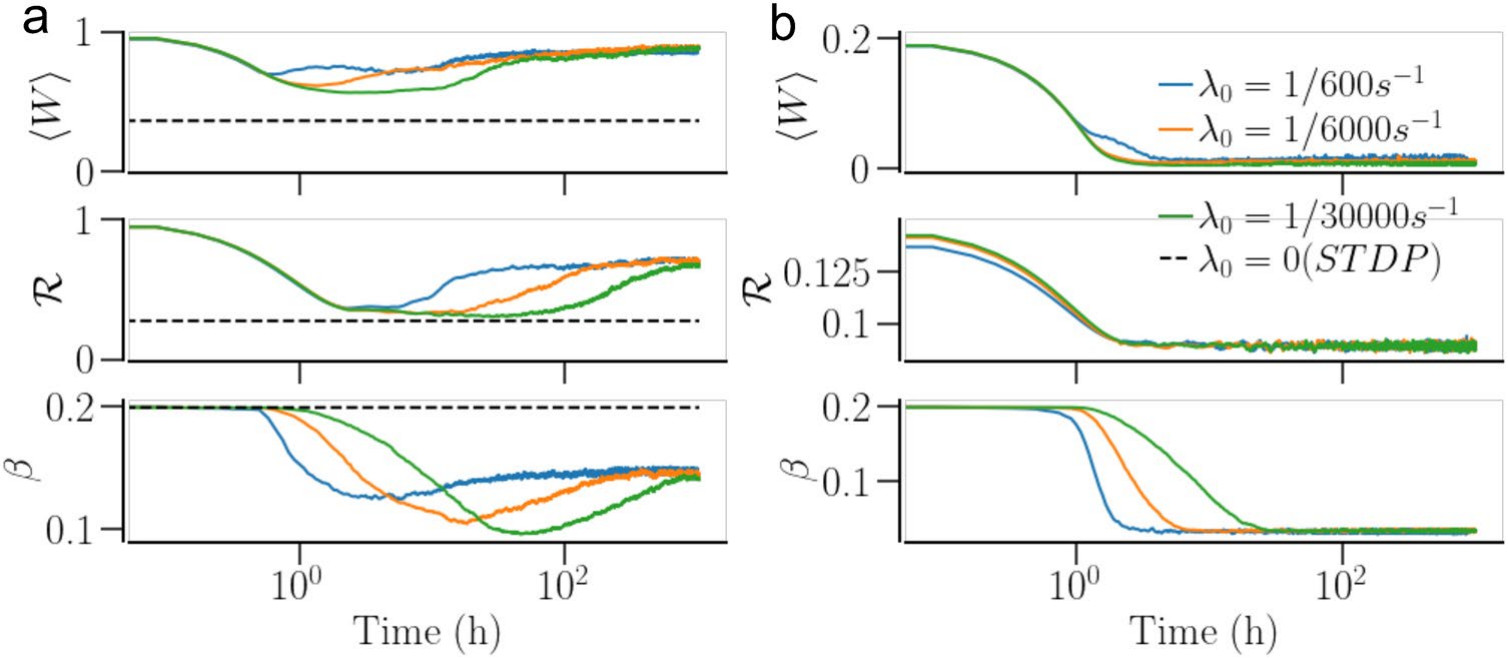
Time evolution of the dynamical measures with different pruning rates (and hence different embodiments of SP) for a network of non-identical oscillators. (**a**) Shows the time traces of the mean synaptic weight, $\langle W\rangle$, the order parameter, ${\mathscr {R}}$, and the mean In-NDD, $\beta$, for a synchronized state and (**b**) shows the same for a desynchronized state. The dashed lines show steady-state values of $\langle W\rangle$, ${\mathscr {R}}$, and $\beta$ for the network with STDP only. Note the different scales of the mean synaptic weight and the order parameter in (**a**) and (**b**). Parameters are $a=0.3$, $\gamma =15$.

Furthermore, we examine the effect of the rate of structural change on the asymptotic states of the network with STDP + SP. The weight-dependent pruning rate, $\lambda _0$, determines the rate of structural change as the rates of addition and homeostatic pruning are relative to $\lambda _0$, scaled by a factor of $\eta$. The smaller the $\lambda _0$ the longer is the time scale of structural change. Figure 7 indicates that the decrease of the rate of SP does not alter the asymptotic states, but rather slows down the transients for those. The case of the synchronized state is particularly illustrative, as it further demonstrates the effect of synchronization enhancement due to SP, discussed above. The network with STDP alone (dashed lines, $\lambda _0=0$) settles in a partially synchronized state with the order parameter ${\mathscr {R}}\approx 0.4$. With SP on, the order parameter first decreases, approaching the value corresponding to the network with STDP only until the addition of excess contacts kicks in, ultimately resulting in a stronger synchronization. At the same time, the average network connectivity settles to $\beta =0.15$, which is significantly smaller than its initial value. Variation of the scaling parameter $\eta$ does not alter the dynamics qualitatively as long as $\eta \ll 1$.

### Statistics of network connectivity

**Figure 8:**
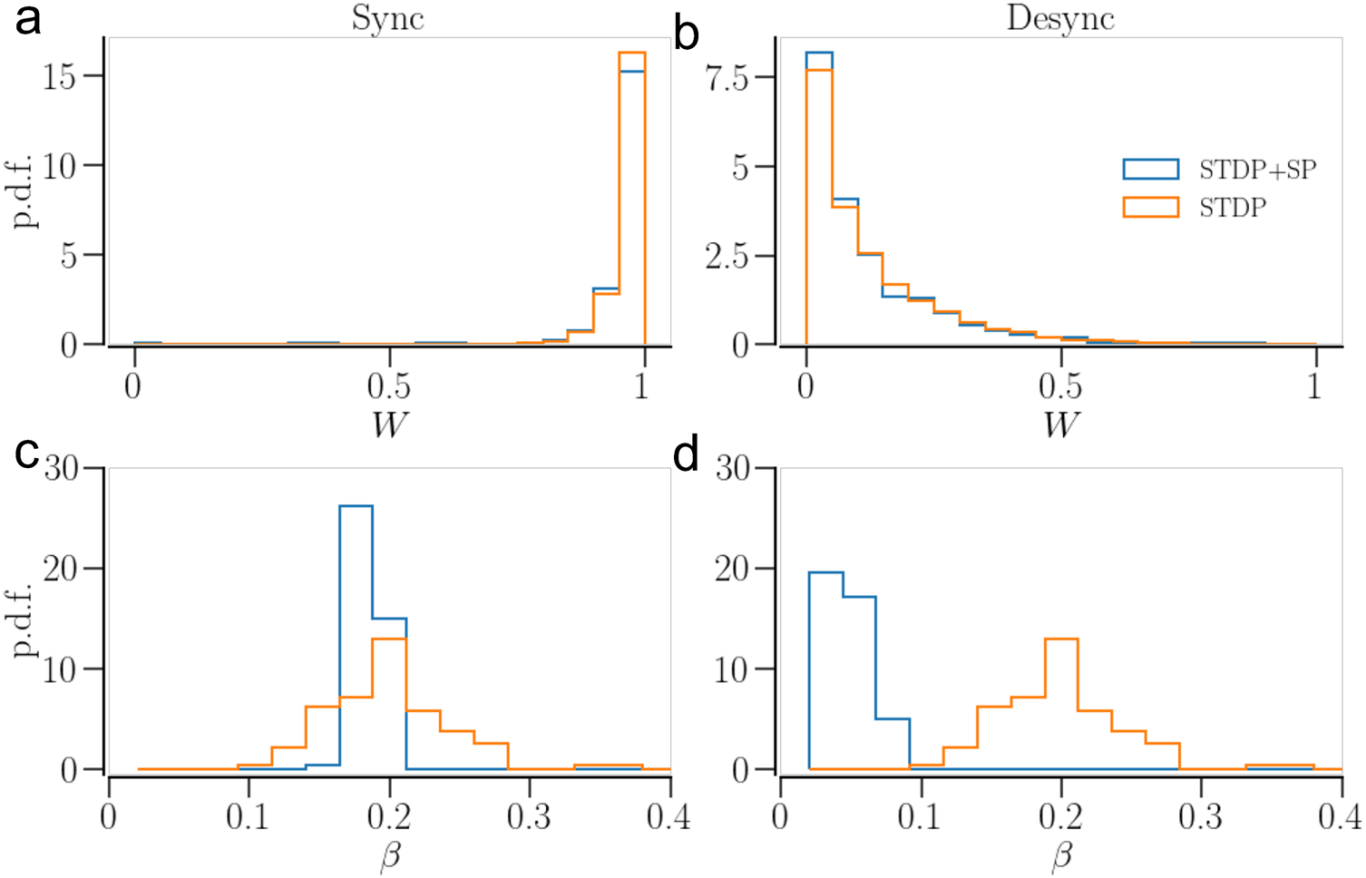
Statistics of synaptic weights and In-NDD for a network of identical oscillators in the asymptotic states belonging to the two plasticity schemes, STDP and STDP + SP. (**a**) and (**b**) show the synaptic weight distributions in a synchronized and a desynchronized state, respectively. (**c**) and (**d**) show the distributions of In-NDD in a synchronized and a desynchronized state, respectively. Parameters are $a = 0.3$ and $\gamma = 3$. Initial conditions for the synchronized sate are $\beta (0)=0.2$ and $\langle W\rangle (0)=1$; and for desynchronized state $\beta (0)=0.2$ and $\langle W\rangle (0)=0.25$.

**Figure 9:**
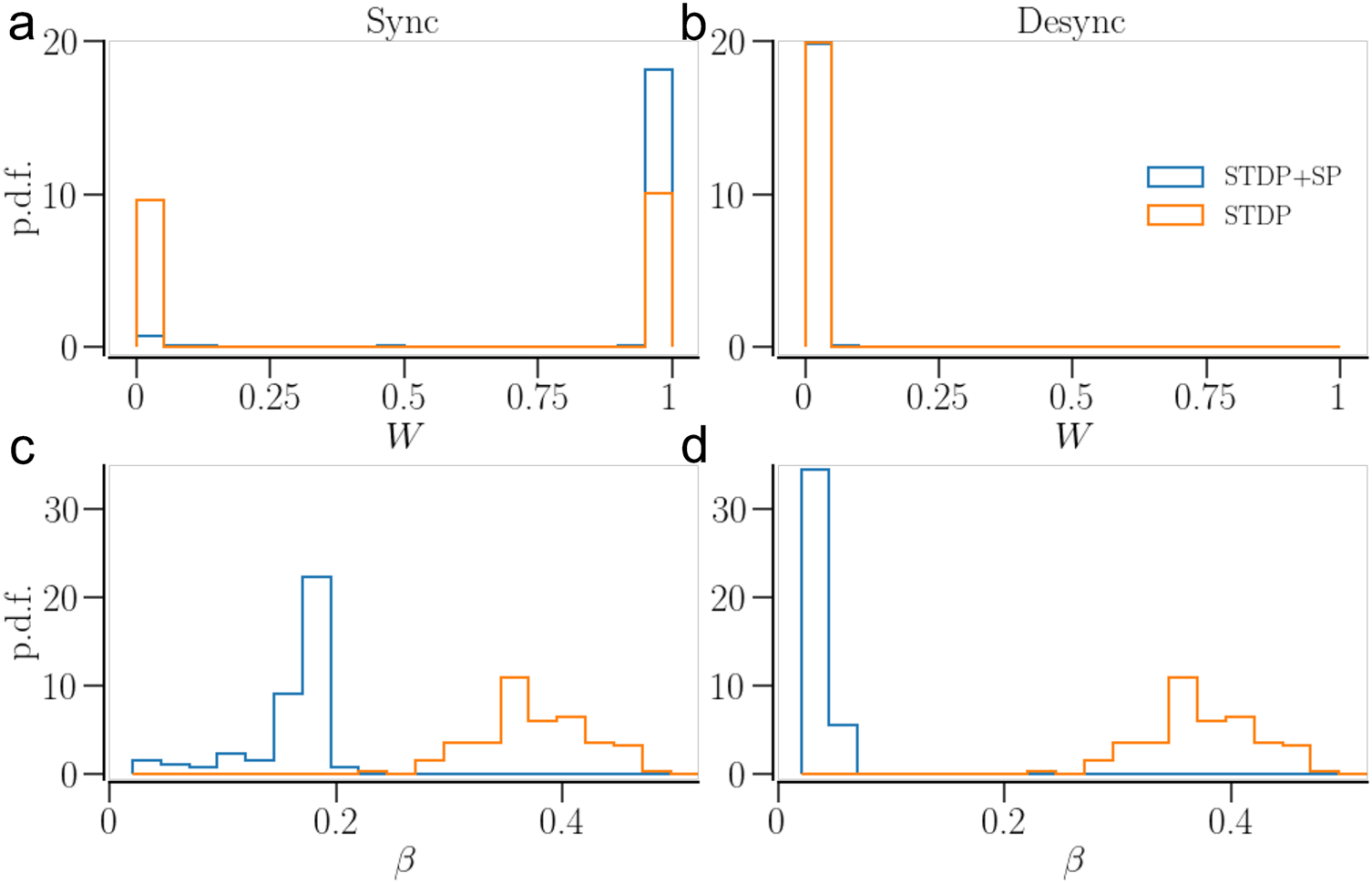
Statistics of synaptic weights and In-NDD for a network of non-identical oscillators in the asymptotic states belonging to the two plasticity schemes, STDP and STDP + SP. (**a**) and (**b**) show the synaptic weight distributions in a synchronized and a desynchronized state, respectively; (**c**) and (**d**) show the distributions of In-NDD in a synchronized and a desynchronized state, respectively. Parameters are $a = 0.3$ and $\gamma = 15$. Initial conditions for the synchronized states are $\beta (0) = 0.4$ and $\langle W\rangle (0)=1$; and for Desync (STDP): $\beta (0)= 0.4$ and $\langle W\rangle (0)=0.06$; for Desync (STDP + SP): $\beta (0)= 0.2$ and $\langle W\rangle (0)=0.2$.

The dynamics of the oscillators together with the plasticity mechanisms determine the statistics of the synaptic weights and the In-NDD of the network. Figures 8 and 9 compare the weight and In-NDD distributions of the asymptotic states in the two plasticity schemes, STDP and STDP + SP, for identical and non-identical oscillator networks, respectively. The initial conditions for the Figures 8 and 9 are such that the networks with STDP only and STDP + SP end up in asymptotic states with similar values of the order parameter.

In the desynchronized state, the synaptic weight dynamics is dominated by LTD, resulting in an overall weight decrease. In a network of identical oscillators, the independent noise may counter weight depression, allowing some synaptic contacts to get potentiated. Hence, a tailed peak is seen at $W \approx 0$ in Fig. 8b for both plasticity schemes. This effect of the temporal noise is smaller for non-identical oscillators, where the heterogeneity of natural frequencies wins over the temporal noise, forcing all the contacts to get depressed, resulting in a sharp peak at $W \approx 0$ in Fig. 9b.

During in-phase synchronization, LTP dominates the synaptic weight dynamics, and hence the overall weight increases. Noise may have a desynchronizing effect and induce depression of some contacts, hence a tailed peak is seen in Fig. 8a for identical oscillators. In a network of non-identical oscillators, the higher frequency oscillators dominate the lower frequency ones^35^, potentiating the contacts from faster to slower oscillators while depressing the others. This leads to two sharp peaks at $W \approx 0$ (smaller) and $W \approx 1$ (larger) for non-identical oscillators in Fig. 9a for the synchronized state.

SP modifies the network structure. The contacts may get pruned (predominantly the weak ones), while new contacts may appear and get potentiated over time. In a desynchronized state, many of the contacts, both previously existing and newly added, become weaker and eventually get pruned, leading to a sparser network. Consequently, the corresponding In-NDD distribution of the network with STDP + SP shifts to smaller values compared to that of the network with STDP only, as shown in Figs. 8, 9d.

**Figure 10:**
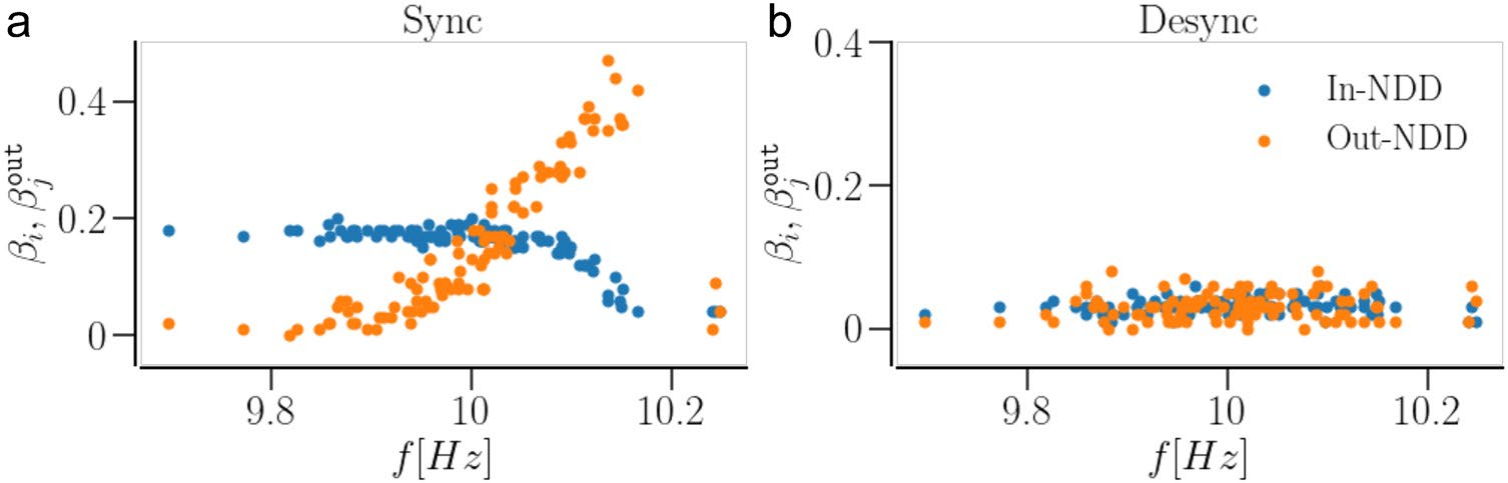
Dependence of oscillator In- and Out- NDD ($\beta _i$ and $\beta _j^\text {out}$, respectively) on the characteristic frequency of the oscillator in a network of non-identical oscillators with STDP + SP, observed in the asymptotic states. (**a**) shows the dependence in a synchronized state and (**b**) shows the same in a desynchronized state. In contrast, for a network with STDP only, the In- and Out-NDD remain independent of the oscillator frequency as the network structure cannot change with activity and remains the same as initially set. Parameters are $a=0.3$, $\gamma =15$; for synchronized state—$\beta (0)=0.4$, $\langle W\rangle =1$; for desynchronized state—$\beta (0)=0.2$, $\langle W\rangle =0.2$.

In transition to a synchronized state, most of the newly added contacts in a network of identical oscillators get potentiated and continue to accumulate until all neurons attain almost the maximum allowed number of presynaptic partners. As a consequence, the In-NDD in Fig. 8c shows a narrow peak at $\beta \approx \beta _\text {max}$. In a synchronizing network of non-identical oscillators, the change in the weight of the newly added contacts depends on the natural frequencies of the connected oscillators, as mentioned above. This results in the accumulation of synaptic contacts, mostly from the faster to the slower oscillators, and the elimination of those going the opposite way. This results in a significant reduction in the total number of contacts in the network even in a synchronized state, evidenced by a shift of the entire In-NDD distribution to smaller values in Fig. 9c. Furthermore, SP leads to the emergence of correlations between the node degrees and the natural frequencies of the oscillators, whereby faster oscillators tend to connect to a larger number of slower oscillators. Accordingly, Fig. 10a shows positive correlations between the Out-NDD and the neurons’ natural frequencies. Such correlations are absent in desynchronized states, Fig. 10b, where the weakly temporally correlated activity leads to no dependence of synaptic weights and node degrees on the natural frequencies. A small cluster of oscillators in Fig. 10a,b with the highest natural frequencies was not in synchrony with the rest of the network. As a result, those oscillators did not develop many contacts with the rest of the network.

### Response to stimulation

The analysis of asymptotic states shows that the remodeling of the network structure due to the presence of SP leads to two main effects. First, in synchronized states, SP removes the unused weak contacts while adding and sustaining strong contacts. This optimizes the network structure for stronger synchronization, compared to the case of STDP only, as indicated by the larger basins of attraction of the synchronized states in Figs. 5, 6a,c. Second, in desynchronized states, the weight-dependent pruning leads to a significantly sparser network. Thus, we expect that compared to the synchronized network with STDP only, a network with STDP + SP is harder to desynchronize, given the same order parameter and mean In-NDD in both plasticity schemes. Conversely, the desynchronized state realized with SP is harder to re-synchronize because of the sparser network structure.

To study the response of a network to a desynchronizing stimulation in the two plasticity schemes, we consider the RVS CR stimulation. The stimulus is applied to the network of identical oscillators, as the order parameter and mean In-NDD in the synchronized state are almost identical for both plasticity schemes, see Fig. 5b,d. Since SP operates on a time scale much longer than STDP, there is no significant change in the network structure during and shortly after stimulation^43^. Thus, the pivotal factor for the desynchronization by RVS CR stimulation is the network structure in the synchronized state, obtained either with STDP + SP or with STDP only.

We parametrize the RVS CR stimulus by its intensity and duration. For both plasticity schemes, a strong and long enough stimulation robustly desynchronizes the network, as demonstrated in Fig. 4b. A long-lasting desynchronization requires shifting the network to the basin of attraction of the desynchronized state, however. On the basins of attraction maps in Fig. 5a,c, this corresponds to a shift from a point in the red region to the blue.

**Figure 11:**
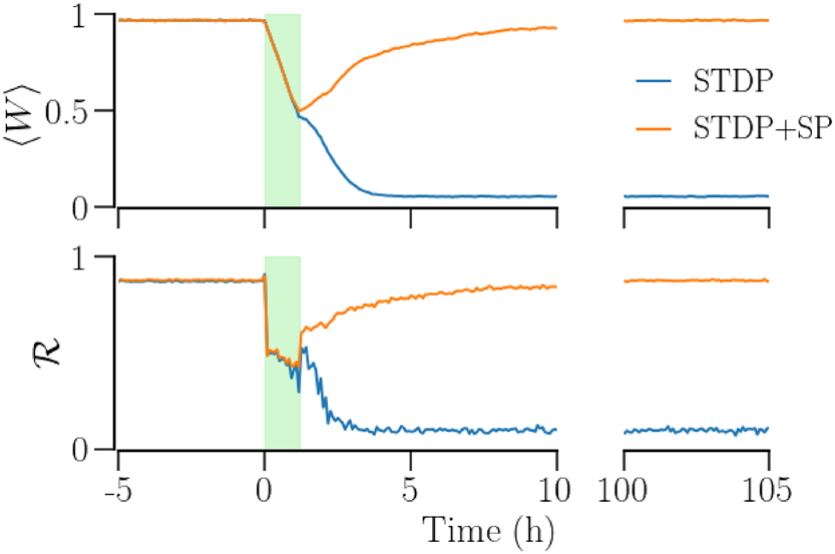
Desynchronization of a network of synchronized identical oscillators using RVS CR stimulation in two plasticity schemes, STDP and STDP + SP. The mean synaptic weight, $\langle W\rangle$, (top) and the order parameter, ${\mathscr {R}}$, (bottom) show the long-term desynchronization of a network with STDP while the network with STDP + SP goes back to synchrony. The shaded region highlights the time interval of stimulation. Parameters are $a=0.3$, $\gamma =3$; stimulation parameters are duration $=70$  min, intensity $I_s=100$, and $F_s=10$ Hz.

The difference in the robustness of the synchronized states realized with the two plasticity schemes is demonstrated by an example in Fig. 11. Using RVS CR with specific duration and intensity, it is only possible to achieve long-lasting desynchronization in the network with STDP alone, while this is not possible in the network with STDP + SP. The order parameter, ${\mathscr {R}}$, the mean synaptic weight, $\langle W\rangle$, and the mean In-NDD, $\beta$, are identical for the initial synchronized states in both plasticity schemes. RVS CR stimulation (shaded region in Fig. 11) desynchronizes the network, indicated by a prompt reduction in ${\mathscr {R}}$, reducing $\langle W\rangle$. By the time the stimulus is removed, the network with STDP alone enters the basin of attraction of the desynchronized state while the one with STDP + SP remains in the basin of attraction of the synchronized state. Accordingly, ${\mathscr {R}}$ and $\langle W\rangle$ remain close to 0 for the network with only STDP, while these measures re-approach the maximum values, ${\mathscr {R}}\approx 1$ and $\langle W\rangle \approx 1$, for the network with STDP + SP.

**Figure 12:**
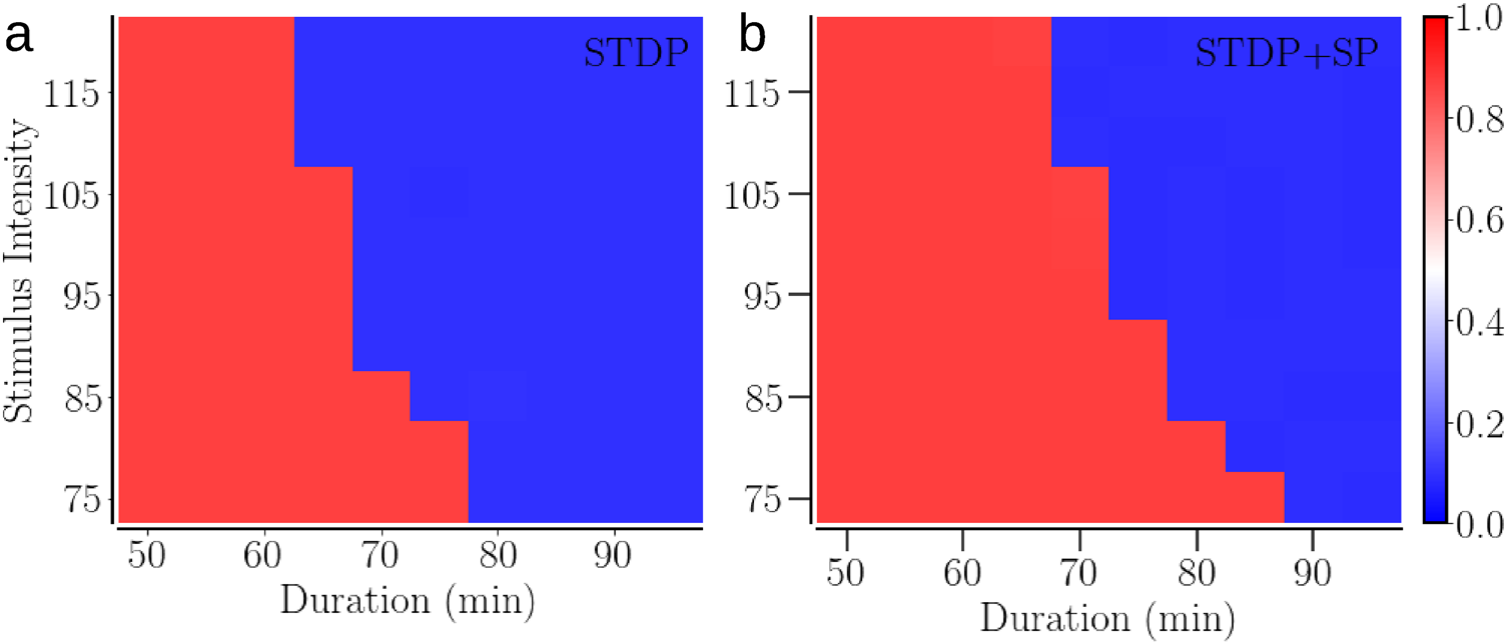
The efficiency of RVS CR stimulation in inducing long-term desynchronization in an initially synchronized network of identical oscillators in the two plasticity schemes—STDP (**a**) and STDP + SP (**b**). Colors indicate the order parameter 100 h after the cessation of stimulation for a given pair of stimulus intensity and duration. The red indicates synchrony and the blue desynchrony. Parameters are $a=0.3$, $\gamma =3$, and $F_s= 10$ Hz.

Varying the stimulus parameters reveals that the synchronized state obtained with STDP + SP is more robust against desynchronizing stimulation, as it requires stronger and longer stimulation, as shown in Fig. 12. The blue areas in that figure correspond to the RVS CR stimulus parameters that result in complete and long-term desynchronization. We confirmed the enhanced robustness of the synchronized state obtained with SP by using a sequential CR stimulus. The ordered stimulation of sites with sequential CR makes the sites fire in a fixed order in every cycle, causing a quicker potentiation of synaptic contacts going from the prior stimulated site to the later and depression of the others. In order to prevent excessive pruning during and soon after stimulation with sequential CR, we reduced the rate of pruning (and hence, SP) to $\lambda _0=1.667\times 10^{-6}$ s$^{-1}$ from $1.667\times 10^{-4}$ s$^{-1}$ used with RVS CR. We further verified the role of the network structure obtained with SP in increasing the robustness of the synchronized state by turning the SP off during and after stimulation, for both sequential CR and RVS CR, so that the difference in response to stimulation arose exclusively from the difference in the network structure in the synchronized state obtained with or without SP. Furthermore, a change in the stimulus frequency produces similar results with both RVS CR and sequential CR. We tested $F_s=7$ Hz and 18 Hz for this purpose.

**Figure 13:**
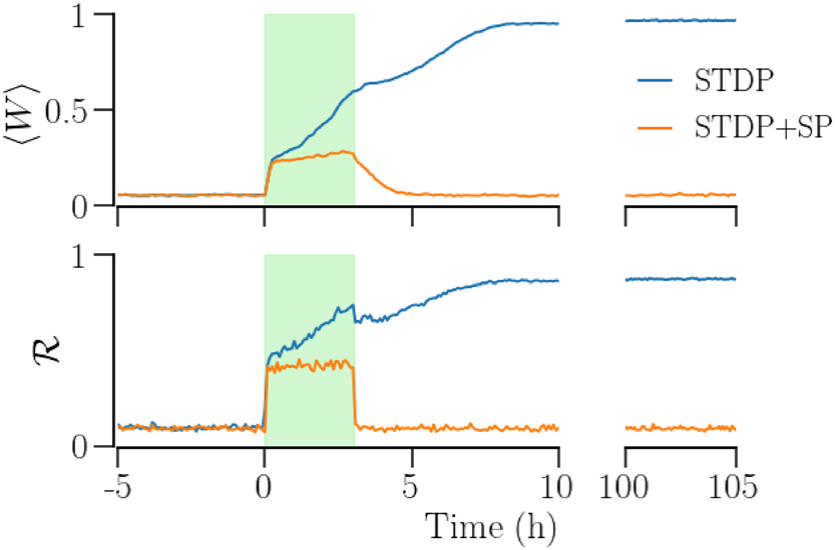
Synchronization of a network of desynchronized identical oscillators by a periodic stimulus in the two plasticity schemes, STDP and STDP + SP. The mean synaptic weight, $\langle W\rangle$, (top) and the order parameter, ${\mathscr {R}}$, (bottom) show the synchronization of a network with STDP while the network with STDP + SP goes back to desynchrony upon cessation of stimulation. The shaded region highlights the time interval of stimulation. Parameter are: $a=0.3$, $\gamma =3$; stimulus parameters are duration $=3$  h, $I_s=100$, and $F_s= 10$ Hz.

External kindling stimulation can bring a desynchronized network to synchrony^35,43^. A synchronizing stimulus potentiates the synaptic weights, eventually leading to global synchronization. However, the desynchronized states realized with SP are characterized by significantly lower number of synaptic contacts than the network with STDP only. Global synchronization requires the building up of synaptic contacts due to SP, which is a slow process. Consequently, a significantly longer and stronger stimulus is required for the synchronization of a desynchronized network with STDP + SP. A representative example is shown in Fig. 13. The desynchronized network with STDP only is amenable to global synchronization by simultaneous stimulation of the sites with a periodic stimulus. However, the stimulus of the same intensity and duration is not adequate for synchronizing the network with STDP + SP. At the cessation of stimulation, the network with STDP only settles in a synchronized state while that with STDP + SP goes back to the desynchronized state.

Stable synchronized and desynchronized states of the network can be thought of as local minima of an effective potential, separated by a barrier. Our results on stimulation of synchronized or desynchronized states of the identical oscillator network suggest that SP deepens both potential wells, rendering both states more robust against stimulation.

For a network of non-identical oscillators, a similar comparison is not as straight forward as above, because SP dramatically alters the average node degrees in both synchronized and desynchronized states. If the network with STDP only is given the same average node degree as that of the network with STDP + SP in its steady synchronized state, then the network with STDP only may possess an order parameter significantly lower than that of the network with STDP + SP, as exemplified in Fig. 7a. Therefore, the network with STDP + SP is harder to desynchronize, compared to the network with STDP only, similar to the case of identical oscillators. However, if the network with STDP only is given a higher average node degree than the network with STDP + SP in a synchronized state such that the order parameters of the two are equal (cf. Figs. 6 and 9), we observe that a synchronized network with STDP + SP, owing to a much smaller average node degree, requires a shorter and weaker stimulation to get desynchronized than that with STDP only. The resynchronization of a desynchronized network of non-identical oscillators shows the same tendency as for identical oscillators. A desynchronized network with STDP + SP may require significantly longer stimulation since the network would need to develop a large number of contacts before it can enter the basin of attraction of a synchronized state.

## Discussion

We studied the dynamics of networks of phase oscillators with two types of plasticity mechanisms that adaptively shape coupling weights and the network structure, modeled by STDP and SP, respectively. With STDP alone, the network can settle in a synchronized or a desynchronized state depending on the initial conditions^12,35^. We consider this system as a minimal model of the neuronal networks where one state corresponds to the physiological mode of operation while the other is associated with abnormal function. For instance, an abnormal synchronization of neurons in cortico-STN networks is observed in patients with PD, while these networks remain in a desynchronized state during normal function^89,90^. On the contrary, in AD, the desynchrony is linked to disease progression while synchrony is desired for normal behavior^23,25–27^.

STDP induces weight adaptation on a longer time scale than the spiking period of the neurons^15,38^. SP adds yet another time scale, which is much longer than that of STDP^15,38^. In this study, we focused on the effects on the steady states caused by adding SP to neuronal phase oscillator networks with STDP. To model key SP features in the framework of a reduced model, we used a simple stochastic model of network rewiring that combined Hebbian weight-dependent pruning with homeostatic pruning and the addition of synaptic contacts, constrained by bounds on the network’s In-NDD.

We showed that SP rewires the network to optimize the synchronized states. The weight-dependent pruning removes the weakest contacts so that they cannot get potentiated by being exposed to random fluctuations. The addition of new contacts that can get potentiated due to STDP, e.g. those going from the faster to slower oscillators, also strengthens synchronization. In the case of identical oscillators, SP leads to a sharp In-NDD distribution, where each oscillator tends to develop the maximum number of presynaptic partners, as demonstrated in Fig. 8c. In the case of non-identical oscillators, SP builds node degree correlations from an initially completely random structure, whereby oscillators with higher natural frequencies tend to have more outgoing and fewer incoming contacts in a synchronized state, as shown in Fig. 10a. As a result, synchronization can be achieved with a smaller number of contacts, i.e., in a sparser network. Consequently, the basins of attraction of synchronized states in Figs.5, 6c extend towards smaller values of initial In-NDD, illustrating the effect of enhanced synchronization for sparser networks due to SP. In the desynchronized state, the SP leads to a network with a minimal number of synaptic contacts, constrained by the lower bound of In-NDD. Self-organization of network structure and the emergence of node degree correlations due to activity-dependent SP were reported in Refs.^91,92^. Our results on optimization of the synchronized state and the emergence of degree-frequency correlations are in agreement with a recent study^14^ that also used a Kuramoto model but with different rules for weight and structural plasticities. A study that employed pruning of low-use connections revealed optimization of distributed routing networks^93^. An examination of the directed network of the suprachiasmatic nucleus (SCN) revealed that the core of SCN has a small incoming and a large outgoing degree, and drives the other neurons in SCN for synchronization^94^.

From PD it is known that abnormally strong neuronal synchrony need not be associated with abnormally up-regulated numbers of synaptic contacts. In fact, the opposite holds, for instance, for the hyperdirect pathway from cortex to STN^95^. Increasing evidence shows that the cortico-STN hyperdirect pathway plays a crucial role for the generation of abnormal neuronal synchrony in PD and displays highly correlated activity between cortex and STN^96–101^. However, the abnormally strong cortico-STN coherence was combined with a significant reduction of the number of cortico-STN synapses^95^. Hence, fewer contacts were associated with strongly coherent neuronal activity.

Studying the spontaneous dynamics and stimulus responses of plastic neuronal networks is relevant for the computational development of stimulation techniques for the treatment of brain disorders characterized by abnormal neuronal synchrony, such as PD^90,102–104^, or by lack of synchrony, such as AD^23,25–27^. In PD, a top-down approach started with comparably simple models, such as phase oscillator networks^35^, in turn advancing to models of increasing complexity^36,43,76^. Already the studies in simpler models like phase oscillator networks enabled to reveal key predictions, such as long-lasting^35,105^ and cumulative^78^ desynchronization effects as well as optimal stimulation patterns^54,106^ and related parameter ranges^107^. In addition, these computational studies revealed stimulus-response characteristics of plastic neuronal networks, highlighting the importance of acute effects (during stimulation), acute after-effects (shortly after cessation of stimulation) and long-term after-effects^54,87,108^ as well as the differential effects of desynchronizing vs. decoupling stimulation protocols^37^. These effects and phenomena were very different from what was known about regular deep brain stimulation. In particular, the computationally derived findings were key to the development of appropriate study protocols for animal experiments^109–112^ and clinical studies^45,113,114^.

Plastic phase oscillator and neuronal networks are typically bistable or multistable with different amounts of synchrony^12,35^, representing physiological and abnormal states in brain disorders characterized by abnormal extent of neuronal synchrony. A bistable neuronal network that can exist in either of the stable synchronous or desynchronous states can be represented by an effective double well potential, where the wells correspond to the two stable states, such as the one shown in Fig. 2 of Ref.^85^. Our results obtained in the model presented here indicate that SP may deepen both the wells, forming more robust synchronous and desynchronous states. This may have a significant impact on the design of therapeutic stimulation and dosage protocols. Specifically, our findings demonstrate that the amount of desynchronization achieved during stimulation need not be sufficient for predicting the dosage required to induce long-term desynchronization by shifting the system to a desynchronized attractor (Fig. 11). In fact, in comparison to a synchronized state of a network of identical oscillators with STDP only, a synchronized state formed with STDP + SP required stronger or longer stimulation to achieve long-lasting desynchronization, see Figs.11, 12. However, once a desynchronized state is achieved with STDP + SP and the network becomes sparser, a stronger and longer synchronizing stimulation is needed to bring the network back to synchrony. More sophisticated dosage regimens, e.g., involving the spacing (pausing) principle^115^ or dedicated test stimuli, might enable us to reduce and/or predict the amount of stimulus dosage required for effective long-term desynchronization. In a previous computational study in a single-compartment conductance-based model of the subthalamic nucleus (STN) and globus pallidus external (GPe) with STDP, an SP mechanism was included that homeostatically adapted the STN neurons’ firing rates to a set point-type target firing rate by generating or pruning synapses^43^. In that study, an epoch of CR stimulation was assumed to favorably decrease the target firing rate, which in turn, made SP decrease the number of synaptic contacts and, hence, ultimately increased the desynchronizing effects of CR stimulation after long epochs without stimulation. The increase of desynchronizing CR effects after sufficiently long stimulation-free periods reflected clinical observations^43^. Although the target-frequency based SP mechanism in that study^43^ and the connectivity-based SP mechanism presented here were very different, in both cases, SP induced significant changes in the network’s spontaneous structure and dynamics as well as its responses to synchronizing and desynchronizing stimulation.

Our results with the phase oscillator model and abstract plasticity rules require further verification. In particular, a spiking neuron model, such as leaky integrate and fire, would allow for a more realistic account of STDP with axonal and dendritic delays and, most importantly, with a significant dependence of network firing rate on network connectivity^37^. Further, conductance-based models, such as the model of subthalamopallidal network^116^ could be implemented to study neurodynamics of PD networks, including responses to stimulation^36,43,73,77,117^. Spiking neuron models for nodes would allow the implementation of different activity-dependent SP mechanisms, regulating the firing rates of individual neurons^38,60^, as opposed to SP primarily reflecting connectivity constraints used in the current study. In addition, future studies should also take into account neurons in bursting mode, which is a characteristic feature of PD^118^. In a first approximation, in the context of bursting, a phase oscillator may correspond to a slowly varying current controlling a fast spike generator and, hence, inducing the bursting, see, e.g., Refs.^119–121^. Bursting entails more complex neuronal dynamics and also more complex STDP rules. In a first approximation, motivated by Ref.^122^, in computational studies the timing difference of the burst onsets was used for the STDP-based synaptic weight update^121^. However, the synaptic weight update does not only depend on spike pairs but also, e.g., on the timing of preceding spikes, hence, requiring more complex STDP rules^122–124^. Furthermore, a spatial organization of the network, not considered in the present study, is necessary to consider the dependence of structural changes on the distance between neurons^38^. We predict that SP will tend to enhance synchronization for networks of excitatory periodically firing spiking neurons, as shown here for the phase oscillator model. We also expect the emergence of node degree correlations due to SP. The effect of SP on the efficacy of desynchronization and synchronization stimulation requires further comprehensive study.

## Data Availability

*Accession codes* Codes are available on request. Requests should be sent to K.C. at kanishk.phy@gmail.com.
